# Histamine upregulates the expression of histamine receptors and increases the neuroprotective effect of astrocytes

**DOI:** 10.1186/s12974-018-1068-x

**Published:** 2018-02-13

**Authors:** Jiawen Xu, Xiang Zhang, Qingqing Qian, Yiwei Wang, Hongquan Dong, Nana Li, Yanning Qian, Wenjie Jin

**Affiliations:** 1 Department of Anesthesiology, The First Affiliated Hospital of Nanjing Medical University, 300 Guangzhou Road, Nanjing, Jiangsu 210029 People’s Republic of China; 2 Department of Anesthesiology, Shanghai First People’s Hospital, Shanghai, People’s Republic of China

**Keywords:** Histamine, Astrocytes, Histamine receptors, Inflammatory factors, Glial cell-derived neurotrophic factor

## Abstract

**Background:**

Astrocytes have attracted increasing attention over recent decades for their role in neuroinflammation. Histamine, a major aminergic brain neurotransmitter, has an important influence on the main activities of astrocytes, such as ion homeostasis, energy metabolism, and neurotransmitter clearance. However, little is known about the impact of histamine on astrocyte immunomodulatory function.

**Methods:**

The expression of all known histamine receptor subtypes was examined in primary astrocytes. Then, primary astrocytes were pretreated with selective histamine receptor antagonists and stimulated with histamine. Cellular activation, proinflammatory cytokine production, and expression of neurotrophic factors were assessed.

**Results:**

Astrocytes could constitutively express three histamine receptors (H1R, H2R, and H3R), and these three histamine receptors could be selectively upregulated to varying degrees upon histamine treatment. Histamine also dose-dependently stimulated astrocyte activation and subsequent production of glial cell-derived neurotrophic factor (GDNF), whereas it suppressed the secretion of the proinflammatory factors tumor necrosis factor-alpha (TNF-α) and interleukin-1β (IL-1β). The effects of histamine were completely abolished by either an H1R or H3R antagonist, while an H2R antagonist attenuated the effects partly.

**Conclusions:**

The present study identified the expression of H1R, H2R, and H3R on astrocytes. We also demonstrated that negative regulation of astrocytic TNF-α and IL-1β production and the enhancement of astrocytic GDNF stimulated by histamine were receptor-mediated processes in which all three of the expressed histamine receptors (H1R, H2R, and H3R) were involved. These findings may further clarify the involvement and mechanism of astrocyte activation in neuroinflammation.

**Electronic supplementary material:**

The online version of this article (10.1186/s12974-018-1068-x) contains supplementary material, which is available to authorized users.

## Background

Astrocytes, the most abundant non-neuronal cell population in the central nervous system (CNS), have been conceptualized as an inert scaffold or as housekeeping cells for many years. However, the results of a growing number of studies suggest that this cell population actively modulates immune responses in the CNS [1]. Hence, defining their particular function during the inflammatory process is an important undertaking. These cells appear to play an important role in either the development of protective immune responses or the progression of damaging inflammation in various stages of CNS disease [2]. Mild activation of astrocytes usually exerts neuroprotective effects and ameliorates early symptoms of neurodegeneration. For instance, the release of neurotrophic factors such as glial cell-derived neurotrophic factor (GDNF), brain-derived neurotrophic factor (BDNF), and neurotrophin-3 (NT-3) can promote neuronal survival and maintain synaptic homeostasis [3, 4]. In particular, recent studies suggest that GDNF can also inhibit microglial activation and alleviate neuroinflammation [5–7]. However, strong activation of astrocytes leads to the secretion of large amounts of cytokines, chemokines, reactive oxygen species, and proinflammatory mediators, affecting the cellular state of surrounding cells such as microglia, neurons, and astrocytes themselves, leading to excitotoxicity, neurodegeneration, and apoptosis [8]. Thanks to their multifaceted role in the inflamed CNS, astrocytes are well suited to determine the site, size, and character of the immune response [9]. In this sense, understanding the regulators and related mechanisms involved in astrocyte activation is key in eliminating deleterious effects of this cell population.

The role for histamine as a neurotransmitter and neuromodulator in many basic homeostatic and higher integrative brain functions is already well established. Additionally, histamine is a potent mediator of inflammation and a regulator of innate and acquired immunity [10]. Four histamine receptors have been identified (H1–H4), and three of them (H1–H3) are prominently expressed in the brain [11]. It has been well documented that histamine importantly influences the main activities of astrocytes, such as ion homeostasis, energy metabolism, and neurotransmitter clearance [12]. However, few data are available regarding the interactions of histamine with astrocyte immunomodulatory function. A study by M. Lipnik-Štangelj et al. demonstrated that histamine and interleukin-1β (IL-1β) acted synergistically in the regulation of nerve growth factor (NGF) secretion from glial cells [13]. Similar results were obtained for NGF secretion stimulated by histamine and interleukin-6 (IL-6) [14, 15]. These findings reveal that histamine may influence astrocyte immunomodulatory function via interacting with several cytokines and neurotrophins.

In the present study, we investigated the expression of histamine receptors on astrocytes and the mechanism of the histamine-induced neuroprotective effect of astrocytes.

## Methods

### Reagents

Dulbecco’s modified Eagle’s medium (DMEM), 0.25% trypsin-EDTA solution and fetal bovine serum (FBS) were purchased from Gibco-BRL (Grand Island, NY, USA).

Histamine was purchased from Sigma–Aldrich (St. Louis, MO, USA). The H1R antagonist cetirizine dihydrochloride (cetirizine), the H2R antagonist ranitidine hydrochloride (ranitidine), and the H3R antagonist carcinine ditrifluoroacetate (carcinine) were purchased from Tocris Bioscience (Bristol, UK). WST-8 dye, RIPA buffer, and a BCA kit were purchased from Beyotime (Shanghai, China). Fluoroshield mounting medium with 4′,6-diamidino-2-phenylindole (DAPI), specific rabbit polyclonal anti-GDNF antibody, and specific rabbit monoclonal anti-H3 receptor antibody were purchased from Abcam (HongKong, China). Rat IL-1β Immunoassay Kit and Rat TNF-α Immunoassay Kit were obtained from R&D Systems, Inc. (Minneapolis, MN, USA). Mouse anti-glial fibrillary acidic protein (GFAP) monoclonal antibody was purchased from Cell Signaling Technology (Boston, MA, USA). Specific rabbit polyclonal anti-H1 receptor and rabbit polyclonal anti-H2 receptor antibodies were purchased from Alomone Labs Ltd. (Israel), and rabbit polyclonal anti-H4 receptor antibody was purchased from Santa Cruz (Santa Cruz Biotechnology, USA). Anti-glyceraldehyde 3-phosphate dehydrogenase (GAPDH) was purchased from Bioworld Technology, Inc. (USA). Anti-rabbit and anti-mouse secondary antibodies were purchased from Jackson ImmunoResearch Laboratories Inc. (Boston, MA, USA). FITC-conjugated goat anti-rabbit IgG and PE-conjugated goat anti-mouse antibodies were purchased from BD Bioscience (USA).

### Astrocyte-enriched cultures

Rat primary astrocytes were prepared according to a previously described protocol with slight modifications [16, 17]. Briefly, whole brains were isolated from postnatal (P1–P2) Sprague–Dawley rats. The meninges and blood vessels were removed completely in cold phosphate-buffered solution. Then, the brains were minced with sterile scissors and digested with 0.25% trypsin-EDTA solution for 10 min at 37 °C. The trypsinization was stopped by adding an equal volume of culture medium, which was high-glucose DMEM containing 10% FBS. The dissociated cells were passed through a 100-μm pore mesh, pelleted at 1500 rpm for 5 min, and resuspended in culture medium. The cell suspension was seeded on cell culture flasks precoated with poly-d-lysine, and the cells were then cultured at 37 °C in a humidified atmosphere of 5% CO_2_/95% air. The culture medium was changed every 3 days. After the glial cells formed a confluent monolayer (10–14 days), the astrocytes were separated from the microglia by shaking. The cultures were passaged into new 10-cm dishes at least three times, 2 weeks apart, to achieve highly pure astrocyte cultures. Immunostaining of the primary cultured cells with antibodies against GFAP confirmed that more than 95% of the cells were astrocytes.

### Cell viability

Cell viability was measured using the dye WST-8 according to the manufacturer’s instructions. Briefly, the astrocytes were seeded into 96-well plates at a density of 3 × 10^4^ cells/well. Following this treatment, WST-8 was added to each well, then the cells were incubated at 37 °C for 2 h and the absorbance was determined at 450 nm using a microplate reader.

### TNF-α and IL-1β assay

Production of TNF-α and IL-1β in the supernatant of the culture medium was measured with ELISA kits (R&D Systems, Minneapolis, MN, USA) according to the manufacturer’s instructions.

### Western blotting

Cells were collected and homogenized in ice-cold lysis buffer. After incubation for 20 min on ice, the cell lysate was centrifuged and the protein concentration in the extracts was measured using a BCA kit. Proteins (50 μg) in cell extracts were denatured with sodium dodecyl sulfate (SDS) sample buffer and separated by 10% SDS–polyacrylamide gel electrophoresis. After electrophoresis, proteins were electrotransferred onto polyvinylidene difluoride (PVDF) membranes (Millipore, Bedford, MA, USA). The blots were blocked with 5% nonfat dry milk dissolved in Tris-buffered saline with TWEEN 20 (TBST) (pH 7.5, 10 mM Tris–HCl, 150 mM NaCl, and 0.1% TWEEN 20) for 1 h at room temperature, then incubated with different antibodies overnight at 4 °C. The following primary antibodies were used: rabbit polyclonal anti-H1 receptor and rabbit polyclonal anti-H2 receptor (1:200), rabbit monoclonal anti-H3 receptor (1:1000), rabbit polyclonal anti-H4 receptor (1:200), rabbit monoclonal anti-GFAP (1:1000), rabbit polyclonal anti-GDNF (1:250), and rabbit monoclonal anti-GAPDH (1:1000). After the membranes were incubated with goat anti-rabbit secondary antibody (1:5000) for 1 h, the protein bands were detected with an enhanced chemiluminescence kit. The relative density of the protein bands was obtained by densitometry using Image Lab software (Bio-Rad, Richmond, CA, USA) and quantified using NIH ImageJ software (Bethesda, MD, USA).

### Immunofluorescence

To evaluate the activation of the astrocytes and the expression of histamine receptors on the astrocytes, we first fixed the cells with 4% paraformaldehyde for 30 min. Unspecific binding was blocked by incubating the cells in a 5% BSA and 0.1% Triton X-100 solution for 1 h at room temperature. Astrocytes were incubated with mouse anti-GFAP monoclonal antibody (1:300) along with rabbit polyclonal anti-H1R, anti-H2R, anti-H4R, and rabbit monoclonal anti-H3R antibodies in the blocking solution overnight at 4 °C. After three washes with PBS, the astrocytes were incubated with the corresponding FITC-conjugated goat anti-rabbit IgG (1:200) and PE-conjugated goat anti-mouse IgG (1:200) at 37 °C for 1 h, and the nuclei were stained with DAPI. Fluorescent images were acquired using a confocal microscope.

### RNA purification and real-time PCR

Total RNA was extracted from primary astrocytes cell cultures using TRIzol Reagent (Invitrogen), and reverse transcription was performed from 1 μg of total RNA for each sample using Transcription First Strand cDNA Synthesis Kits (Roche) according to the manufacturer’s instructions. Real-time PCR amplification was performed using the StepOne Real-Time PCR Detection System (Foster City, CA) with SYBR Green master mix (Applied Biosystems, Foster City, CA) in a final volume of 10 μl that contained 1 μl of cDNA template from each sample. All the primers used for qRT-PCR were obtained from GeneCopoeia (USA). The cycling conditions were 95 °C for 30 s followed by 40 cycles of 95 °C for 5 s and 60 °C for 30 s. The relative mRNA values were normalized to the beta-actin gene as an internal control and calculated using the comparative cycle threshold (ΔΔCt) method.

### Statistical analysis

The values shown are mean ± s.e.m. The significance of the difference between control and samples treated with various compounds was determined by one-way ANOVA followed by the post hoc least significant difference test. Differences were considered significant at *p* < 0.05.

## Results

### Selective expression of histamine receptors H1R, H2R, and H3R but not H4R in primary astrocytes

**Fig. 1:**
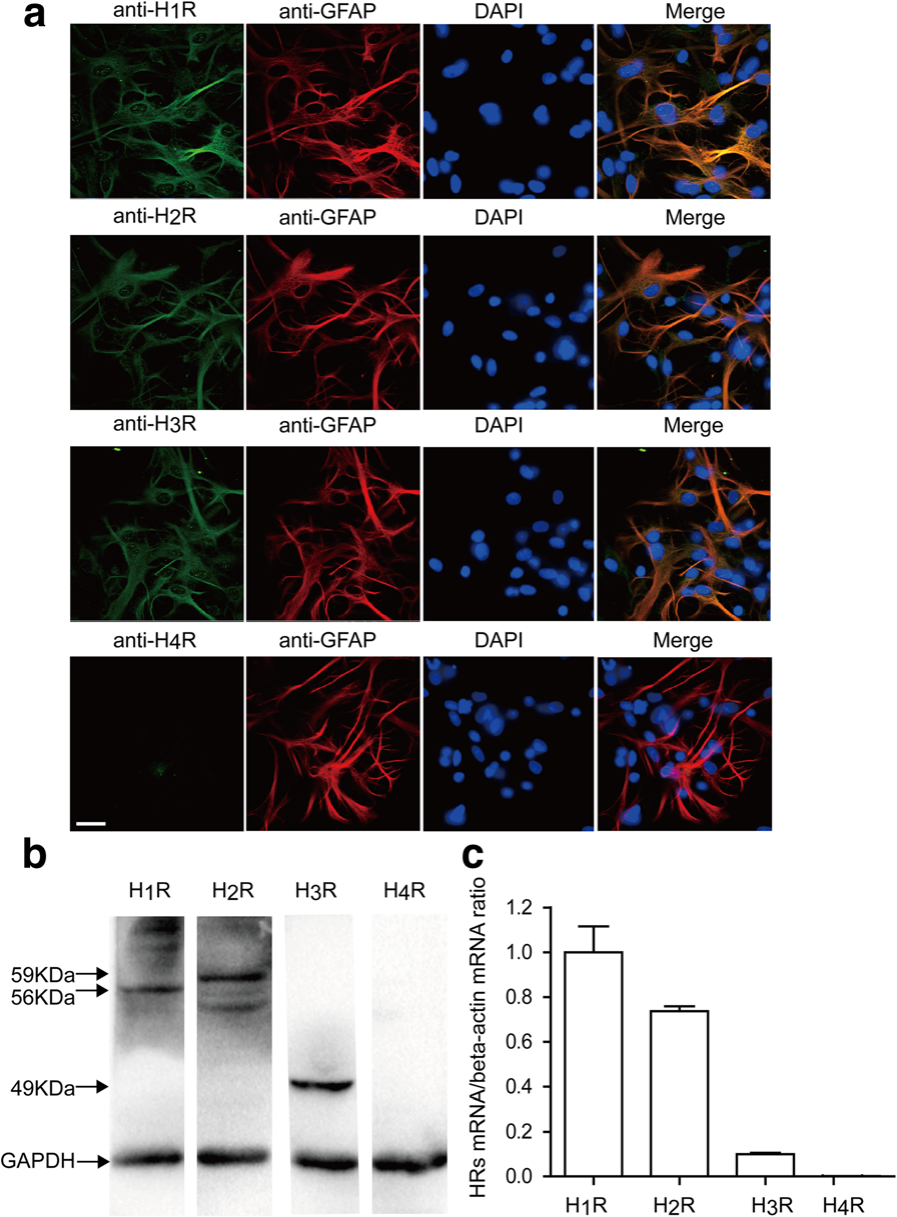
Identification of expression of histamine receptors in primary cultured astrocytes. **a** Immunolocalization of H1R, H2R, H3R, and H4R in astrocytes was performed by using antibodies against H1R, H2R, H3R, and H4R (green) and an antibody against the astrocyte marker GFAP (red). The results were imaged with a laser scanning confocal microscope. The blue staining represents DAPI. Scale bar = 25 μm. **b** Western blotting analysis of H1R, H2R, H3R, and H4R in the extracts of rat astrocytes. **c** The expression levels of the histamine H1, H2, H3, and H4 receptor subtypes were examined by quantitative RT-PCR. The data are presented as the mean ± s.e.m. of three independent experiments

Numerous studies have demonstrated that cultured astrocytes from different mammalian brain regions express H1 and H2 receptors to varying degrees [18–21]. Notably, recent evidence has established the existence of H3 receptors on astrocytes [4, 22]. However, to date, little is known of the expression of H4 receptors in primary astrocytes. In the present study, we performed double-antigen immunofluorescence staining using astroglial cell-specific GFAP and each of four histamine receptors. The immunofluorescence data showed the expression of the histamine receptors H1R, H2R, and H3R but not H4R in the primary astrocytes (Fig. 1a). As shown in Fig. 1b, Western blotting analysis revealed prominent bands at the expected molecular weights of 56, 59, and 49 kDa in the astrocytic cell extract, representing H1R, H2R, and H3R respectively. However, the expected H4 band did not appear. Consistent with the results above, histamine H4 receptor mRNA was not detected using real-time PCR (Fig. 1c). In addition, the expression levels indicated that the quantities of H2 and H3 receptor mRNA were 0.7 and 0.1 times the quantity of H1 receptor mRNA, respectively.

### Histamine-induced astrocyte activation

**Fig. 2:**
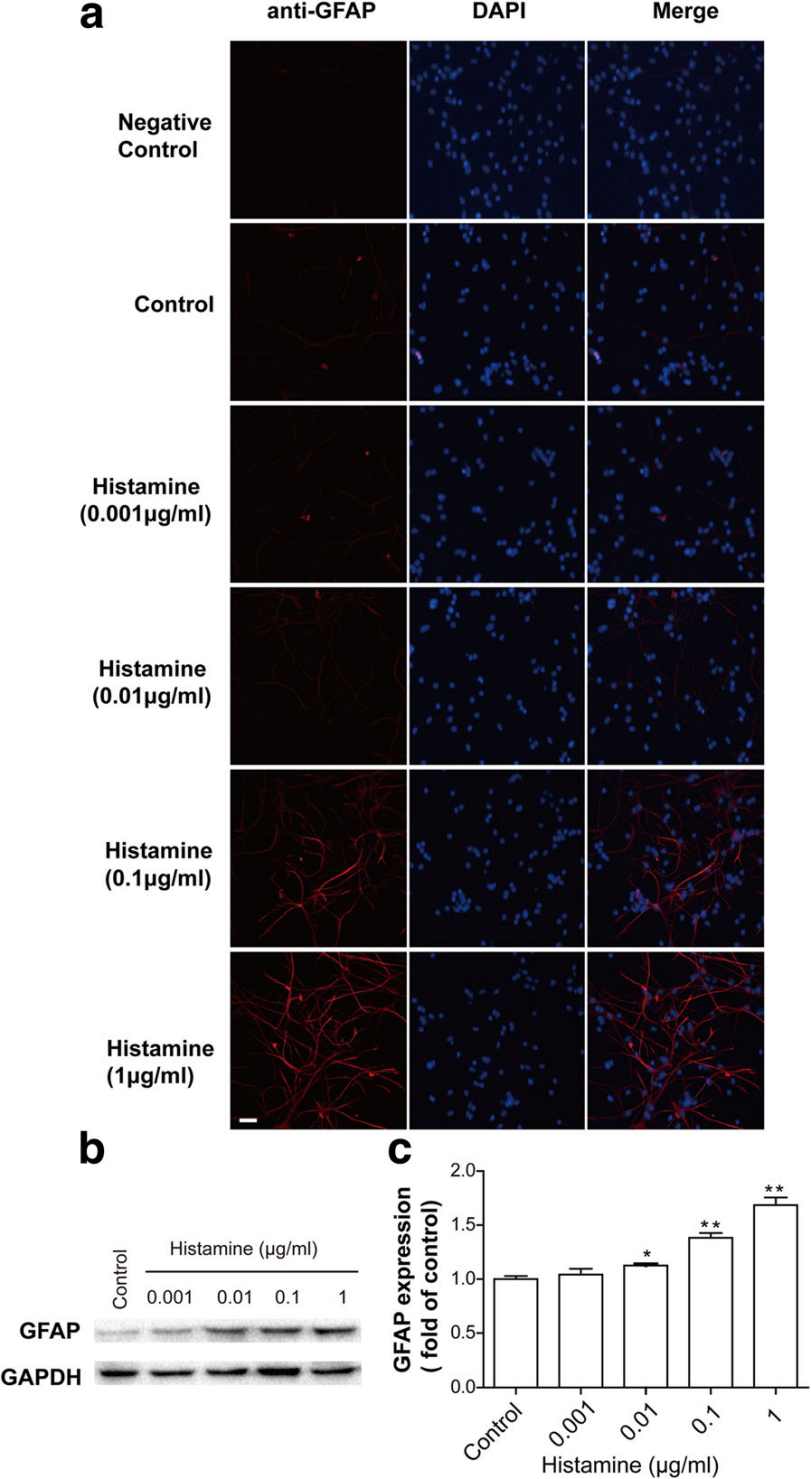
Histamine-induced astrocyte activation. Primary astrocytes were treated with histamine (0.001–1 μg/ml) for 24 h. **a** Cells were stained with GFAP antibody, and upregulated GFAP expression (red) on activated astrocytes was observed using confocal microscopy. The blue staining represents DAPI. Scale bar = 50 μm. **b** and **c** Levels of GFAP were detected by Western blotting, quantified, and normalized to GAPDH levels. Values are expressed relative to the control, which was set to 1. *p < 0.05, **p < 0.01 versus the control group. The data are presented as the mean ± s.e.m. of three independent experiments

A WST-8 cell survival assay revealed that incubation with histamine (0.001, 0.01, 0.1, and 1 μg/ml) for 24 h had no effect on astrocyte viability (see Additional file 1). Activated astrocytes were detected by their GFAP expression levels. After incubation with different doses of histamine (0.001–1 μg/ml) for 24 h, GFAP expression (in red) was greatly upregulated (Fig. 2a). The elevated expression of GFAP induced by histamine was further validated by Western blotting (Fig. 2b, c). These results suggest that histamine can activate astrocytes.

### Histamine upregulated the expression of histamine receptors in astrocytes

**Fig. 3:**
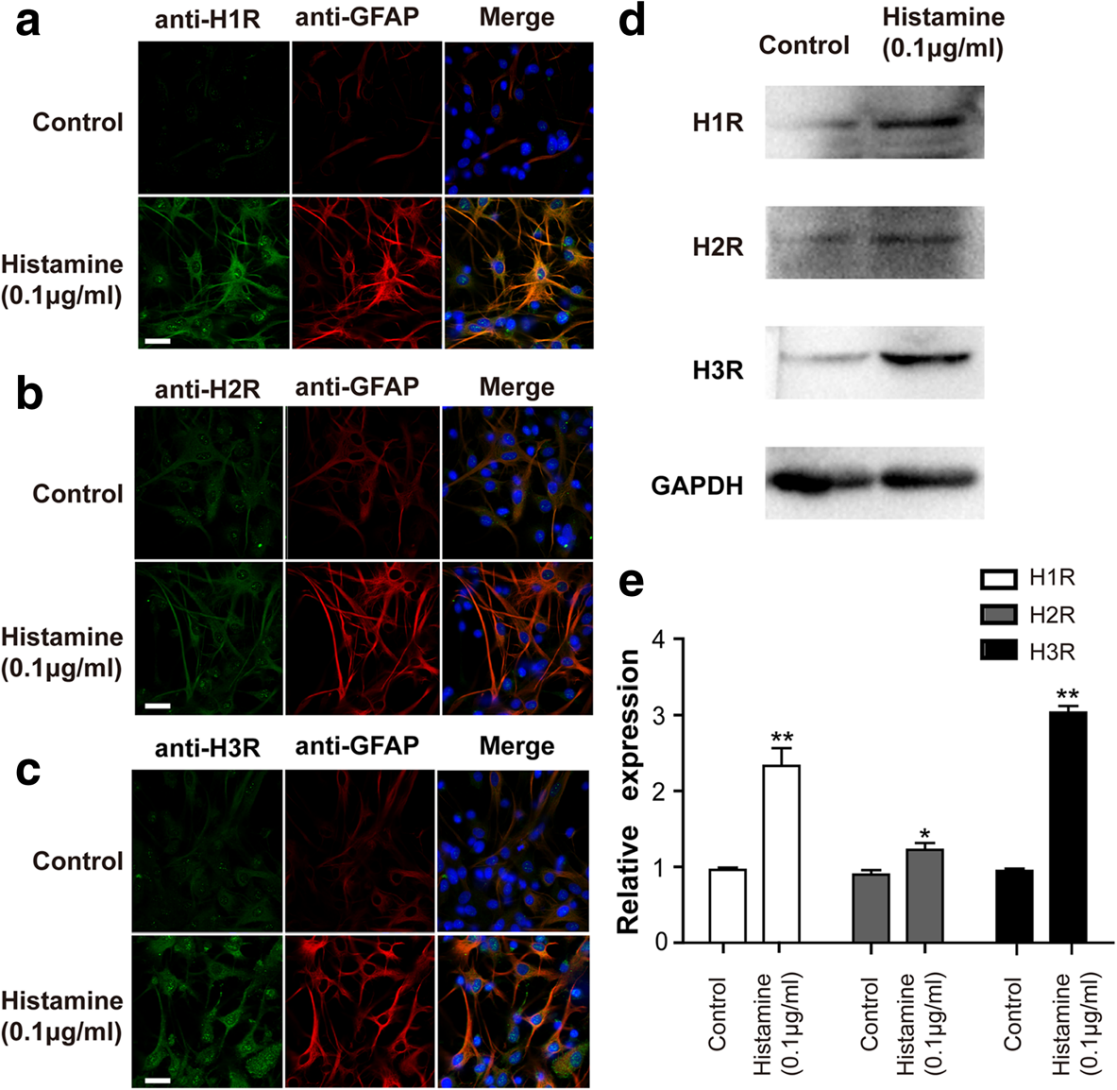
Upregulation of H1R, H2R, and H3R expression in primary cultured astrocytes by histamine. Primary astrocytes were stimulated with histamine at 0.1 μg/ml for 24 h. **a**–**c** Immunofluorescence analysis of GFAP and histamine receptor expression. The cells were stained for the astrocyte marker GFAP (red) and for H1R, H2R, and H3R (green). Upregulated GFAP and HR expression on activated astrocytes was observed using confocal microscopy. The blue staining represents DAPI. Scale bar = 25 μm. **d** The expression levels of the histamine H1, H2, and H3 receptor subtypes were detected via Western blotting using specific antibodies. Each blot is representative of three experiments. **e** The expression levels of the histamine H1, H2, and H3 receptor subtypes were examined by quantitative RT-PCR. *p < 0.05, **p < 0.01 versus the control group. The data are presented as the mean ± s.e.m. of three independent experiments

To ascertain whether histamine modulates the expression of the H1R, H2R, and H3R proteins in astrocytes, we employed immunofluorescence in the present study. Given the notable increase of astrocyte GFAP expression (in red) in response to histamine challenge (0.1 μg/ml), we concurrently evaluated the expression of these three histamine receptors. The results showed that histamine (0.1 μg/ml) provoked significant upregulation of the expression of H1R and H3R in primary cultured astrocytes. Meanwhile, no obvious increase in H2R expression was found (in green). These observations were further supported by Western blotting analysis (Fig. 3d). Similarly, as shown in Fig. 3e, following the incubation with histamine (0.1 μg/ml) for 24 h, the mRNA expression levels of H1R, H2R, and H3R increased to approximately 233, 123, and 303% of the control values, respectively.

### Effects of HR antagonists on histamine-induced suppression of astrocytic TNF-α and IL-1β production

**Fig. 4:**
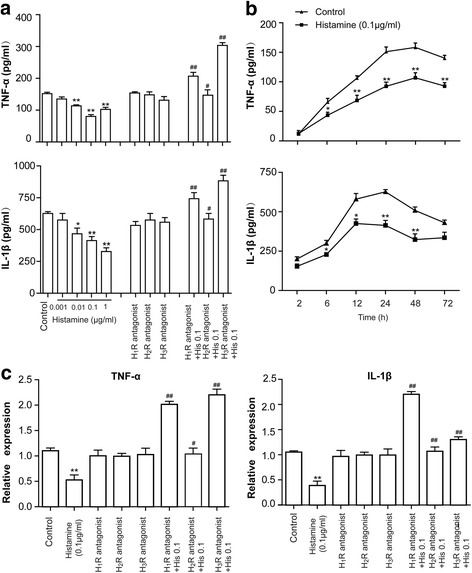
Effects of HR antagonists on HA-induced suppression of astrocytic TNF-α and IL-1β production. **a** Incubation with histamine at the doses of 0.001, 0.01, 0.1, and 1 μg/ml for 24 h produced a concentration-dependent suppression of TNF-α and IL-1β secretion from primary cultured astrocytes. The H1R antagonist cetirizine (10 μM), the H2R antagonist ranitidine (10 μM), and the H3R antagonist carcinine (10 μM) were added to astrocytes alone or 30 min before addition of histamine (0.1 μg/ml) for 24 h. **b** Time courses of suppression of TNF-α and IL-1β release by histamine. Histamine at 0.1 μg/ml was incubated with astrocytes at 37 °C for 2, 6, 12, 24, 48, and 72 h. **c** The levels of TNF-α and IL-1β mRNA expression were analyzed by quantitative RT-PCR. *p < 0.05, **p < 0.01 versus the control group. ^#^*p* < 0.05, ^##^*p* < 0.01 versus histamine (0.1 μg/ml) treatment groups. The data are presented as the mean ± s.e.m. of three independent experiments

Because astrocytes take part in the intracerebral immune response by secreting proinflammatory mediators, the levels of proinflammatory mediators were determined in the present study. As shown in Fig. 4a, histamine dose-dependently decreased TNF-α secretion from primary cultured astrocytes. Similarly, the IL-1β level was significantly declined after treatment with histamine. The time course study (incubation with 0.1 μg/ml histamine for 6, 12, 24, and 48 h) also showed that histamine could suppress the production of TNF-α and IL-1β by astrocytes (Fig. 4b). While the H1R antagonist cetirizine (10 μM), the H2R antagonist ranitidine (10 μM), and the H3R antagonist carcinine (10 μM) separately failed to affect the production of TNF-α and IL-1β in astrocytes, they diminished the effect of histamine (0.1 μg/ml) on TNF-α and IL-1β generation in astrocytes. Notably, the TNF-α and IL-1β decrease induced by histamine (0.1 μg/ml) was reversed by the H1R antagonist, the H3R antagonist, and partly by the H2R antagonist, suggesting that the H1R and the H3R antagonists had a greater effect on TNF-α and IL-1β release than the H2R antagonist. At the same time, the mRNA expression levels of H1R, H2R, and H3R did not have significant changes upon antagonist treatment, suggesting that antagonist treatment has little effect on receptor expression (Additional file 2). These results indicated that all three histamine receptors (H1R, H2R, and H3R) participated in the histamine-induced suppression of TNF-α and IL-1β secretion from astrocytes. However, H1R and H3R are likely to play dominant roles in this process.

### Effects of HR antagonists on histamine-stimulated GDNF protein expression in astrocytes

**Fig. 5:**
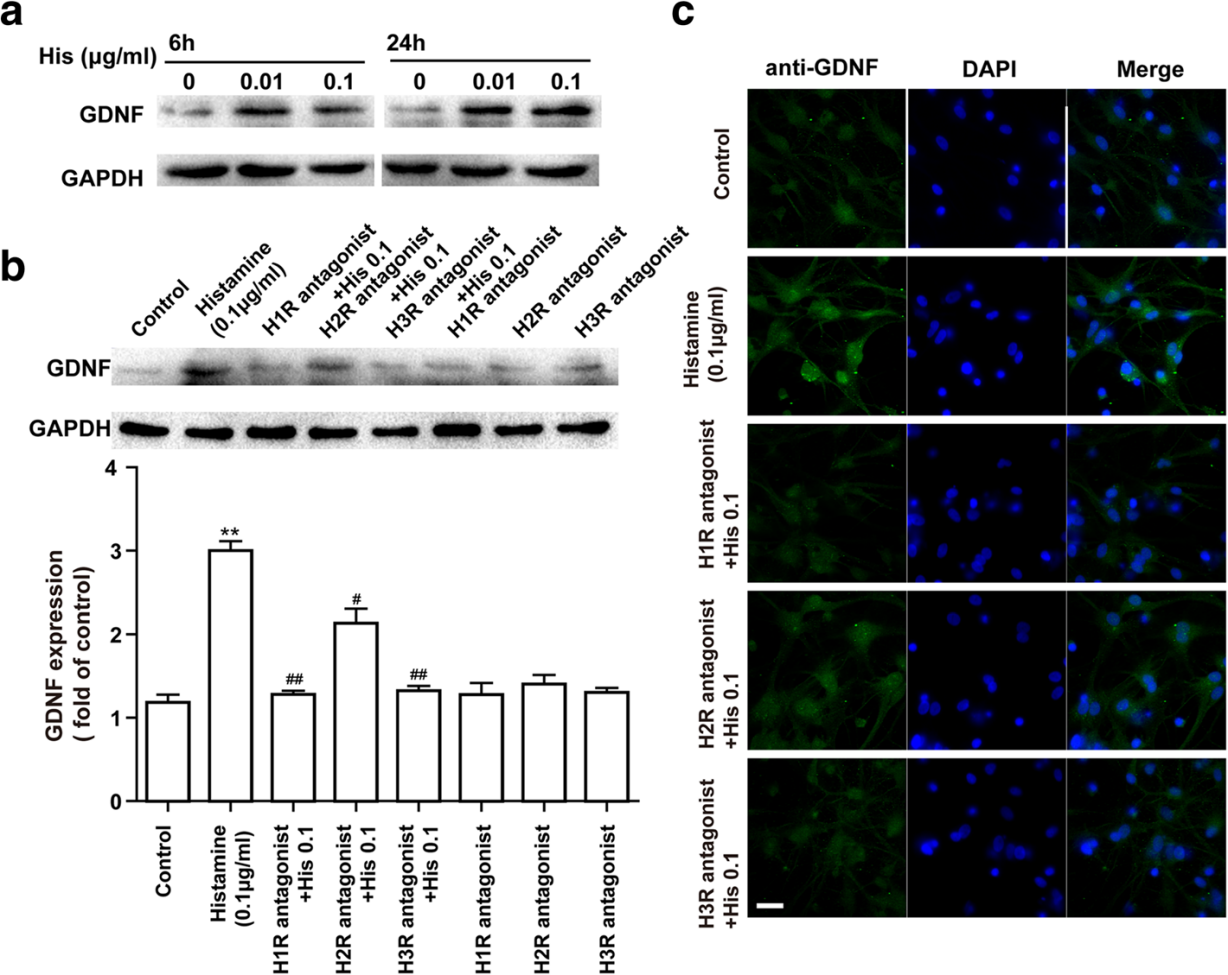
Effects of HR antagonists on HA-induced stimulation of GDNF protein levels in astrocytes. **a** Incubation with histamine at the doses of 0.01 and 0.1 μg/ml for 6 and 24 h significantly promoted GDNF expression in astrocytes. **b** Astrocyte cells were stimulated with histamine (0.1 μg/ml) in the absence or presence of the H1R antagonist cetirizine (10 μM), the H2R antagonist ranitidine (10 μM), and the H3R antagonist carcinine (10 μM) added 30 min before stimulation. At 24 h after the addition of histamine, levels of GDNF were detected by Western blotting, quantified, and normalized to GAPDH levels. Values are expressed relative to the control, which was set to 1. **p < 0.01 versus the control group. ^#^*p* < 0.05, ^##^*p* < 0.01 versus histamine (0.1 μg/ml) treatment groups. The data are presented as the mean ± s.e.m. of three independent experiments. **c** The astrocyte cells were stained with anti-GDNF antibody. Expression of GDNF (green) in activated astrocytes was visualized by confocal microscopy. The blue staining represents DAPI. Scale bar = 25 μm

Interestingly, a protective effect was achieved upon negative regulation of astrocytic TNF-α and IL-1β production in response to histamine. Based on the recent observation that GDNF plays an important role in limiting neuroinflammation [7], we next examined the expression of GDNF in astrocytes upon histamine treatment. As expected, after 6 and 24 h of treatment, histamine at 0.01 and 0.1 μg/ml markedly promoted GDNF secretion from astrocytes (Fig. 5a). As shown in Fig. 5b, the increase in GDNF expression induced by histamine (0.1 μg/ml) was almost completely blocked by either the H1R or the H3R antagonist, while the H2R antagonist partly attenuated the stimulatory effect of histamine. However, the H1R antagonist cetirizine (10 μM), the H2R antagonist ranitidine (10 μM), and the H3R antagonist carcinine (10 μM) separately did not have impact on the secretion of GDNF from astrocytes. These Western blot findings were confirmed by immunofluorescence (Fig. 5c).

## Discussion

Histamine plays a central role in innate and acquired immunity: in allergy and inflammation, it is closely associated with mast cell function; in immunomodulation and autoimmunity, it regulates T cell function [10]. Histamine has a diverse effect on many cell types due to differential expression of its receptors. In recent years, astrocyte-associated neuroinflammation has attracted considerable attention. However, little is known about the role of histamine in astrocyte activation and related brain inflammation. In this study, we provided evidence that astrocytes express H1, H2, and H3 but not H4 receptors. In addition, histamine was able to selectively upregulate expression of these three histamine receptors and to induce astrocyte activation. Furthermore, by triggering H1, H2, and H3 receptors, histamine suppressed the production of TNF-α and IL-1β and stimulated the synthesis of GDNF by astrocytes. Therefore, our results established that negative regulation of astrocytic TNF-α and IL-1β production along with upregulation of GDNF synthesis is a mechanism by which histamine may evoke the neuroprotective effect of astrocytes.

Inflammation plays a part in most, if not all, CNS insults. Microglia, described as brain-resident phagocytes, are well established as early sensors of damage and recruiters of multicellular inflammation [23]. In addition, astrocytes are now emerging as cells that can exert either potent proinflammatory functions or crucially protective anti-inflammatory functions, as regulated by specific signaling inputs [24]. Previous studies have demonstrated that histamine, contained not only in neurons but also in brain mast cells, is responsible for the overactivation of microglia and the excessive release of proinflammatory mediators from activated microglia [25, 26]. To examine whether histamine is also a mediator of astrocyte activation, we designed the present study. Given that astrocyte reactivity was originally characterized by morphological changes (hypertrophy, remodeling processes) and overexpression of the intermediate filament protein GFAP, we examined the level of GFAP to evaluate the activity of astrocytes and found that histamine could induce astrocyte activation in a dose-dependent fashion. Unexpectedly, our further experiments showed that the levels of proinflammatory cytokines from astrocytes did not positively correlate with the expression of GFAP. Indeed, concentration-dependent inhibition of astrocytic TNF-α and IL-1β production was observed in the presence of histamine at 0.01, 0.1, and 1 μg/ml. Consistent with the result above, a study by Huszti et al. demonstrated that histamine could attenuate the increased production of astrocytic TNF-α induced by stream stress or IL-1β [27]. While knowledge about the effect of histamine on astrocytic immunomodulatory function is limited, we suggest that histamine could suppress the secretion of proinflammatory cytokines in astrocytes to reduce neuroinflammation.

Astrocyte restriction of cytotoxic CNS inflammation is a recent discovery. Essential anti-inflammatory roles of astrocytes have now been demonstrated in diverse models of CNS injury and disease [28]. Through the secretion of reparatory neurotrophic factors, moderate astrocyte activation plays a crucial role in the recovery of the injured CNS [29]. Previous studies have revealed the stimulatory effect of histamine on the synthesis of two neurotrophins, NGF and NT-3, in astrocytes, and such effect is thought to contribute to promoting neuronal survival and maintaining synaptic homeostasis [4, 30]. In addition to NGF and NT-3, astrocytes have the ability to produce GDNF, and our results showed that its expression was greatly enhanced in the presence of histamine. It was recently reported that GDNF could inhibit microglial activation and neuroinflammation both in vivo and in vitro [6, 7]. Thus, GDNF may be a possible mediator of the anti-neuroinflammatory effect of histamine. Taken together, these results indicated that attenuation of the proinflammatory effects of astrocytes and improvement of their anti-inflammatory responses seems to be important mechanisms underlying the protective effects of histamine.

Histamine triggers its pleiotropic effects by activating one or several histamine receptors on different cells. To date, four subtypes of receptors (HR1, HR2, HR3, and HR4) have been identified [31, 32]. Our study confirmed the expression of histamine receptors H1R, H2R, and H3R, but not H4R, in the primary astrocytes, which appeared to support the notion that the expression of H4R is limited to neuronal cells and microglia [33–35] (Additional file 3). Moreover, detailed gene transcripts of HRs in rat astrocytes revealed that the mRNA expression level of native H3R is low compared with H1R and H2R, but following incubation with histamine at 0.1 μg/ml for 24 h, the mRNA expression level of H3R increased to approximately 303% of the control value, while H1R and H2R rose to 233 and 123% respectively. The H1R subtype has been found to be connected to most astrocytic functions, such as ion homeostasis, energy metabolism, neurotransmitter clearance, and neurotrophic activity, which are regulated by histamine [12]. The H2R subtype is associated with histamine-induced glycogen breakdown via increases in cAMP formation [36]. Although less well studied, the H3R subtype, the newest member of the histamine receptor family identified on astrocytes, is implicated in inducing expression and synthesis of NT-3 in cultured astrocytes [4]. In the present study, we found that all three of these histamine receptors (H1R, H2R, and H3R) were involved in the histamine-driven suppression of TNF-α and IL-1β secretion and induction of GDNF synthesis in astrocytes. However, H1R and H3R appeared to play dominant roles in these two processes. Histamine induces local inflammation reactions either by direct action on target cells or by indirect influence, in which it activates other humoral and/or cellular effector systems. As shown in this study, increased production of GDNF and decreased secretion of proinflammatory cytokines in astrocytes exposed to histamine have been found to coincide temporally. Additionally, they changed in the opposite directions on pretreatment with an H1R or H3R antagonist. Whether astrocyte-derived GDNF, in turn, plays a role in the suppression of astrocytic proinflammatory cytokine production needs further study.

As a main source of histamine, mast cells in CNS have been demonstrated to take part in the pathogenesis of experimental autoimmune demyelinating diseases, experimental allergic neuritis, and experimental autoimmune encephalomyelitis (EAE) [37]. Mast cells (MCs) are primary effector cells of the innate immune system and the “first responders” to injury, rather than glial cells. MCs and their secreted mediators modulate inflammatory processes and can thereby either contribute to neurological damage or confer neuroprotection [38, 39]. We have previously reported that activated MCs can trigger astrocyte activation and subsequent production of inflammatory cytokines in vitro, indicating that activated MCs led to a proinflammatory profile in astrocytes [17]. In the present study, we found that histamine (0.001, 0.01, 0.1, and 1 μg/ml) was inclined to exert neuroprotective and anti-inflammatory effects on astrocytes. However, the impact of histamine at higher concentrations is not known. Tryptase, the major secretory protein of mast cells, was found to modestly reduce intracellular ROS production at lower concentrations but significantly increase TNF-α and IL-6 secretion at higher concentrations in astrocytes [40]. Taken together, the evidence shows that astrocytes play multifaceted roles in the healthy and injured CNS, which are determined in a context-specific manner by diverse signaling events that vary with the nature and severity of different CNS insults. On the other hand, our findings above are now limited to in vitro studies, more in vivo studies and detailed work is required to address the issue further.

## Conclusions

In summary, to our knowledge, this is the first study to verify the exact expression of histamine receptors in astrocytes and demonstrate the ability of histamine in upregulation of H1R, H2R, and H3R expression in those cells. Furthermore, by triggering H1, H2, and H3 receptors, histamine suppressed the production of TNF-α and IL-1β and stimulated the synthesis of GDNF by astrocytes. These results suggest that histamine might play an important role in astrocyte activation and neuroinflammation-related diseases, which further clarifies the involvement and mechanism of astrocyte activation in neuroinflammation.

## Additional files

Additional file 1: Figure S1. The effects of histamine and HR antagonists on cell viability in primary astrocytes. (A) The astrocytes were exposed to different concentrations of histamine (0.001–1 μg/ml) for 24 h. (B) The astrocytes were exposed to the H1R antagonist cetirizine (10 μM), the H2R antagonist ranitidine (10 μM), and the H3R antagonist carcinine (10 μM) and/or histamine (0.1 μg/ml) for 24 h. Cell viability was determined using a colorimetric method. Each data point represents the mean ± s.e.m. of at least three separate experiments in which treatments were performed in quadruplicates. (TIFF 507 kb)

Additional file 2: Figure S2. The effects of HR antagonists on expression levels of the histamine H1, H2, and H3 receptor subtypes. The astrocytes were exposed to the H1R antagonist cetirizine (10 μM), the H2R antagonist ranitidine (10 μM), and the H3R antagonist carcinine (10 μM) for 24 h. The expression levels of the histamine H1, H2, and H3 receptor subtypes were examined by quantitative RT-PCR. The data are presented as the mean ± s.e.m. of three independent experiments. (TIFF 365 kb)

Additional file 3: Figure S3. The expression levels of the histamine H4 receptor subtype in primary microglia and astrocytes. The expression of H4 receptor subtype was detected via Western blotting using specific antibody. The blot is representative of three experiments. (TIFF 546 kb)

## Abbreviations

BDNF: Brain-derived neurotrophic factor
CNS: Central nervous system
GAPDH: Glyceraldehyde 3-phosphate dehydrogenase
GDNF: Glial cell-derived neurotrophic factor
GFAP: Glial fibrillary acidic protein
IL-1β: Interleukin-1β
IL-6: Interleukin-6
MCs: Mast cells
NGF: Nerve growth factor
NT-3: Neurotrophin-3
