# Supplementary figures and images for: Histamine upregulates the expression of histamine receptors and increases the neuroprotective effect of astrocytes
Source: J Neuroinflammation. 2018 Feb 13;15:41. doi: 10.1186/s12974-018-1068-x (PMC5809996; doi:10.1186/s12974-018-1068-x)

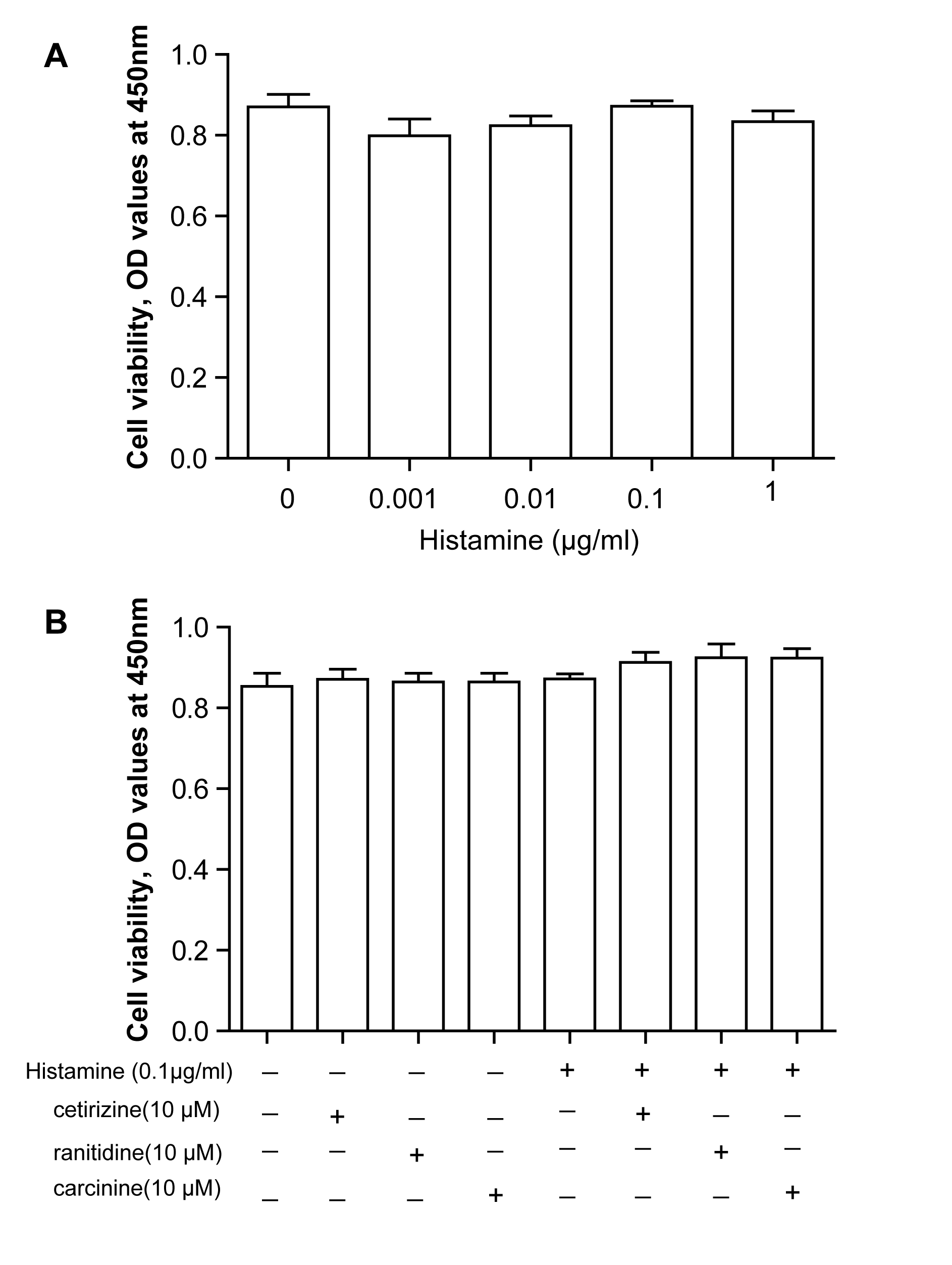

Supplement: Supplementary file 1 — The effects of histamine and HR antagonists on cell viability in primary astrocytes. (A) The astrocytes were exposed to different concentrations of histamine (0.001–1 μg/ml) for 24 h. (B) The astrocytes were exposed to the H1R antagonist cetirizine (10 μM), the H2R antagonist ranitidine (10 μM), and the H3R antagonist carcinine (10 μM) and/or histamine (0.1 μg/ml) for 24 h. Cell viability was determined using a colorimetric method. Each data point represents the mean ± s.e.m. of at least three separate experiments in which treatments were performed in quadruplicates. (TIFF 507 kb) [file 12974_2018_1068_MOESM1_ESM.tif]

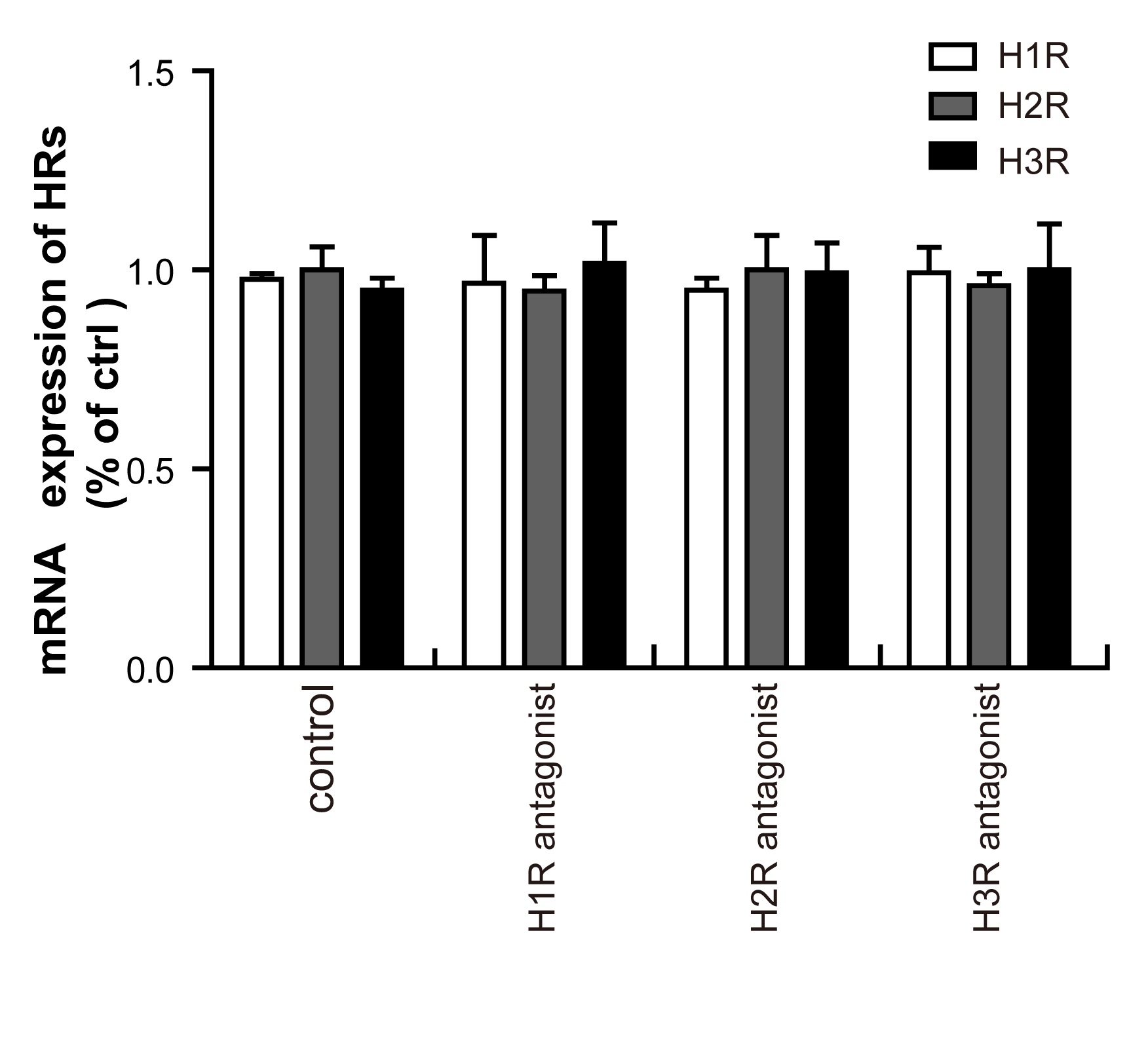

Supplement: Supplementary file 2 — The effects of HR antagonists on expression levels of the histamine H1, H2, and H3 receptor subtypes. The astrocytes were exposed to the H1R antagonist cetirizine (10 μM), the H2R antagonist ranitidine (10 μM), and the H3R antagonist carcinine (10 μM) for 24 h. The expression levels of the histamine H1, H2, and H3 receptor subtypes were examined by quantitative RT-PCR. The data are presented as the mean ± s.e.m. of three independent experiments. (TIFF 365 kb) [file 12974_2018_1068_MOESM2_ESM.tif]

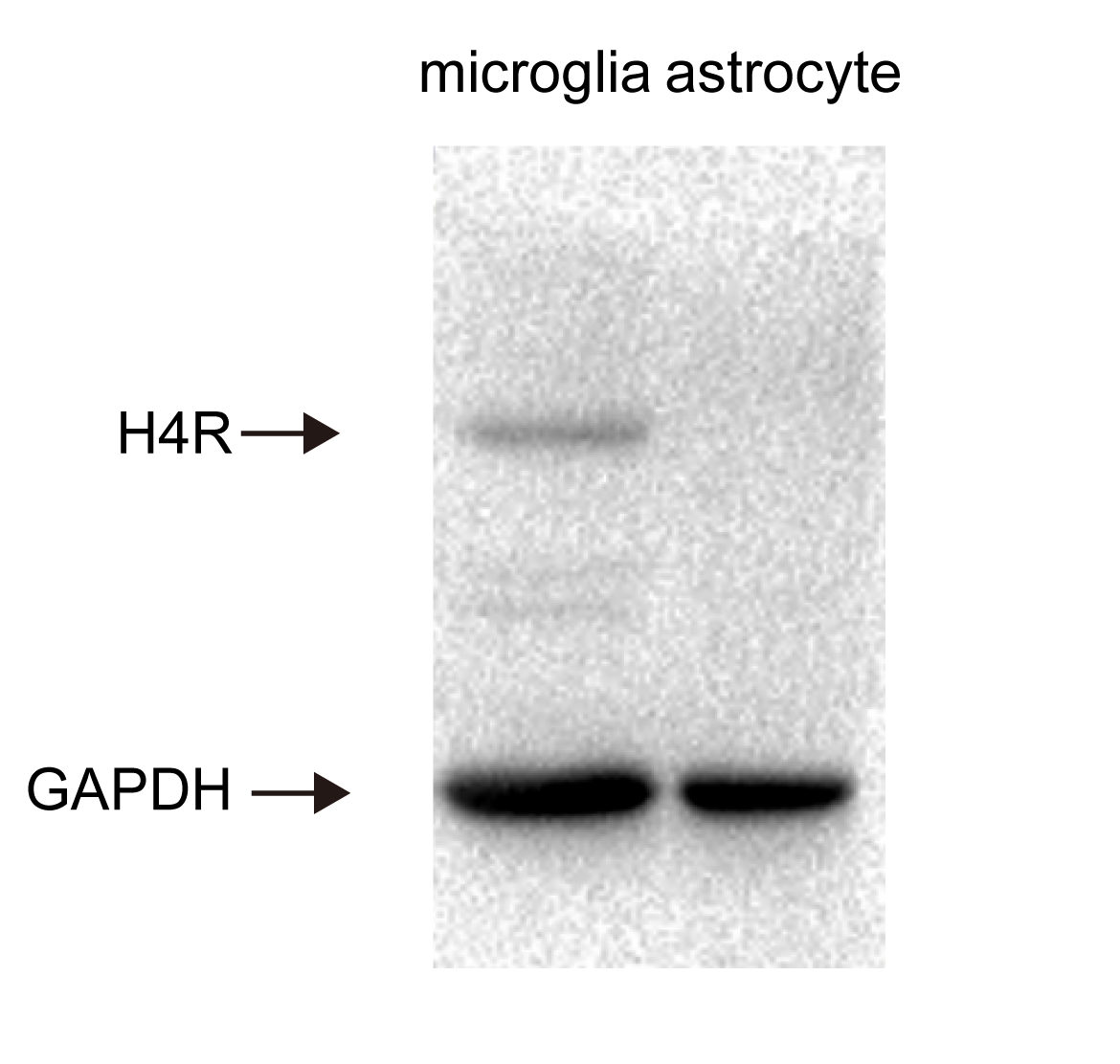

Supplement: Supplementary file 3 — The expression levels of the histamine H4 receptor subtype in primary microglia and astrocytes. The expression of H4 receptor subtype was detected via Western blotting using specific antibody. The blot is representative of three experiments. (TIFF 546 kb) [file 12974_2018_1068_MOESM3_ESM.tif]
